# The MOSAIC Initiative: Addressing Multimorbidity Among Black Americans Through Community-Engaged Research

**DOI:** 10.1093/geroni/igaf122.1821

**Published:** 2025-12-31

**Authors:** Courtney Thomas Tobin, Angela Gutierrez, Roland Thorpe

## Abstract

Multimorbidity, the co-occurrence of two or more chronic conditions, disproportionately affects Black Americans and remains an urgent public health challenge. The M.O.S.A.I.C. (Multimorbidity Outcomes & Solutions for African/Black Americans in California) Initiative is a community-engaged research collaborative dedicated to understanding and addressing multimorbidity among Black populations. This session brings together studies that examine key determinants of multimorbidity and strategies for intervention, providing a comprehensive foundation for the MOSAIC Initiative’s efforts. Yan’s study leverages data from the California Health Interview Survey to explore life course patterns of multimorbidity, highlighting age- and gender-specific disparities that call for targeted prevention efforts. Robinson’s study examines the role of socioeconomic status in shaping multimorbidity, demonstrating that higher SES does not consistently translate to better health for Black Americans, particularly Black men. Mills’ study investigates how perceived neighborhood racial composition influences multimorbidity, revealing significant age-specific associations that underscore the importance of place-based interventions. Finally, the MOSAIC Initiative’s own research identifies best practices in health communication strategies for addressing multimorbidity among Black Los Angeles residents, emphasizing the need for tailored dissemination approaches across different age groups. Together, these studies provide critical insights into the structural, social, and environmental factors contributing to multimorbidity in Black communities. By integrating these findings, the MOSAIC Initiative aims to develop evidence-based, community-driven solutions to reduce multimorbidity disparities and improve health outcomes for Black Americans.

